# Editorial: Exploring nutrigenomics in managing metabolic inflammation

**DOI:** 10.3389/fnut.2026.1970649

**Published:** 2026-09-21

**Authors:** Omar Ramos-Lopez, Gabriela Batitucci, Ibrahim AlZaim

**Affiliations:** 1 Medicine and Psychology School, Autonomous University of Baja California, Tijuana, Baja California, Mexico; 2 Laboratory of Cell Signaling, Obesity and Comorbidities Research Center, University of Campinas, Campinas, Brazil; 3 Department of Biomedicine, Aarhus University, Aarhus, Denmark; 4 Steno Diabetes Center Aarhus, Aarhus University Hospital, Aarhus, Denmark

**Keywords:** gene expression, metabolic inflammation, nutriepigenetics, nutrigenetics, nutrigenomics, personalized nutrition

Metabolic inflammation is a chronic, low-grade systemic condition that contributes to the development of several diseases, including obesity, type 2 diabetes, and cardiovascular disease. Advances in nutrigenomic sciences have provided important insights into the pathophysiological processes that drive metabolic inflammation, creating opportunities to design and implement customized nutritional strategies. Disciplines such as nutrigenetics, nutrigenomics, and nutriepigenetics are helping to decode the complex biological interplay between diet and gene interactions in inflammation. To support a comprehensive exploration of this field, the scope of this Research Topic is defined by its focus on both practical relevance and theoretical advancement in nutrigenomic sciences, with the aim of deepening current understanding of personalized diet-based interventions to mitigate or prevent metabolic inflammation.

Nutrigenetics aims to explore how variations in DNA sequence affect individual responses to diet or nutrients (1). One of the most studied genes in this field is the fat mass and obesity gene (*FTO*), which has been associated with increased susceptibility to obesity. In this Research Topic, Hajira et al. investigated the impact of a high-protein, low-calorie diet (HPLC, about 800 kcal and 100 g protein/d for 4 weeks) on body composition and inflammatory markers in 60 overweight and obese individuals with no comorbidity, and examined whether the *FTO* variant rs9939609 may modify such dietary effects. Effect size estimates revealed significant variation in anthropometric measurements explained by the *FTO* variant, whereby carriers of the AA genotype lost less body weight, BMI, waist circumference, and waist-to-hip ratio compared with participants carrying the TT genotype at the end of the intervention. No genotype effects were found for inflammatory outcomes. The authors concluded that the *FTO* rs9939609 variant modifies the effects of the HPLC diet on adiposity parameters in their population, supporting a link between *FTO* genotype, protein intake, and body composition. Further investigation into the underlying mechanisms driving gene-diet interaction is encouraged, as well as assessment of the efficacy and applicability of this nutritional approach within personalized weight- loss interventions.

Epigenetics examines heritable changes to the genome that are not caused by alterations in the DNA sequence. Examples include DNA methylation, post-translational modifications to histone proteins, and non-coding RNAs such as miRNAs (2). The role of epigenetic modifications induced by dietary interventions in metabolic pathways has attracted considerable interest in recent years. In this context, Wen et al. reviewed the implications of miRNAs in cardiovascular disease associated with the consumption of ultra-processed foods (UPF). They state that UPF overconsumption may cause cardiovascular damage through additive exposure, adverse lipid composition, high glycaemic load, disruption of the food matrix, processing-generated toxicants, and microbiome perturbation. These insults converge on inflammatory and oxidative pathways that regulate miRNA expression in vascular and endothelial cells. This repeated signaling appears to induce miRNAs involved in atherogenesis (miR-21, miR-33, miR-34a, miR-155), macrophage activation (miR-155 and miR-146a), lipid dysregulation (miR-33, miR-122 and miR-30c), vascular aging and plaque vulnerability (miR-34a), and platelet activation and vascular remodeling (miR-223 and miR-21). UPF exposure also reshapes extracellular vesicle (EV)-associated miRNA networks, providing a mechanism by which diet-induced stress in metabolic tissues can be transmitted to the vascular system. Some therapeutic strategies involve circulating miRNA/EV-miRNA biomarkers and miRNA-targeted therapeutics; however, dietary and lifestyle interventions are considered the most scalable approaches for modulating miRNA biology and preventing cardiovascular disease in clinical practice. These strategies include reducing UPF intake and adopting minimally processed dietary patterns, such as Mediterranean or Nordic diets, complemented by regular physical activity and optimized sleep. Regarding the role of epigenetics in liver disease, Wang et al. investigated the effects of polyphenol-rich Chinese olive extracts on lipid accumulation in HepG2 cells and explored the underlying molecular mechanisms, including gene and miRNA expression profiles. The results revealed that extract treatment was accompanied by the downregulation of lipogenesis-related genes and the upregulation of β-oxidation-associated genes, which correlated with the decreased expression of miR-122 and miR-21. The authors concluded that attenuation of lipid accumulation by Chinese olive extracts involved miRNA modulation and lipid-associated changes, supporting the hepatoprotective potential of this nutritional treatment.

Nutrients (including carbohydrates, proteins, fats, vitamins, and minerals) act as important biochemical signals and building blocks that influence gene expression and protein synthesis. Nutrigenomics examines the effect of nutrients on gene expression, which ultimately shapes the proteome and affects the structural and functional state of cells (3). A mechanistic understanding of translational and post-translational regulation in response to different nutrient conditions may help identify potential dietary targets and support innovative therapeutic approaches based on nutritional interventions to improve human health. In this context, Ding et al. used a vitamin D receptor-knockout (*VDRKO*) mouse model to investigate cell-autonomous functions of *VDR* in adipose tissue under vitamin D-deficient conditions, employing high-throughput proteomic technology and parallel reaction monitoring-based targeted proteomics. They found that *VDR* deletion profoundly reprogrammed the adipose tissue molecular landscape in *VDRKO* mice, with increased protein levels involved in metabolic processes, ribosomes, biological regulation, and endoplasmic reticulum activity. In contrast, collagen proteins were downregulated within the protein digestion and absorption network. Further analysis revealed extensive succinylation changes in proteins related to energy metabolism and arginine biosynthesis. The authors conclude that the extensive changes identified in both protein expression and succinylation highlight the complex regulatory pathways controlled by vitamin D signaling in adipose tissue, opening new perspectives for understanding nutrient-related metabolic diseases and offering potential therapeutic strategies.

In summary, the articles in this Research Topic contribute to the knowledge base of nutrigenomic sciences, advancing personalized nutrition in metabolic inflammation. On the one hand, they emphasize the role of genotype in prescribing diets for weight loss and metabolic/inflammatory control. They also explore the epigenetic mechanisms (especially microRNAs) underlying the development of cardiovascular disease associated with the consumption of ultra-processed foods and the lipid-lowering effects of polyphenol-rich olive extracts in the liver. Finally, they highlight the impact of essential nutrient deficiencies, such as vitamin D, on the proteome and its metabolic and immune implications. Future directions in this field include integrating other areas of personalization, such as the gut microbiota and metabolome, to provide a holistic view of human metabolism and develop timely precision nutrition strategies for managing metabolic inflammation. The use of artificial intelligence, bioinformatics, and systems biology tools will help to achieve these goals in the medium term.
